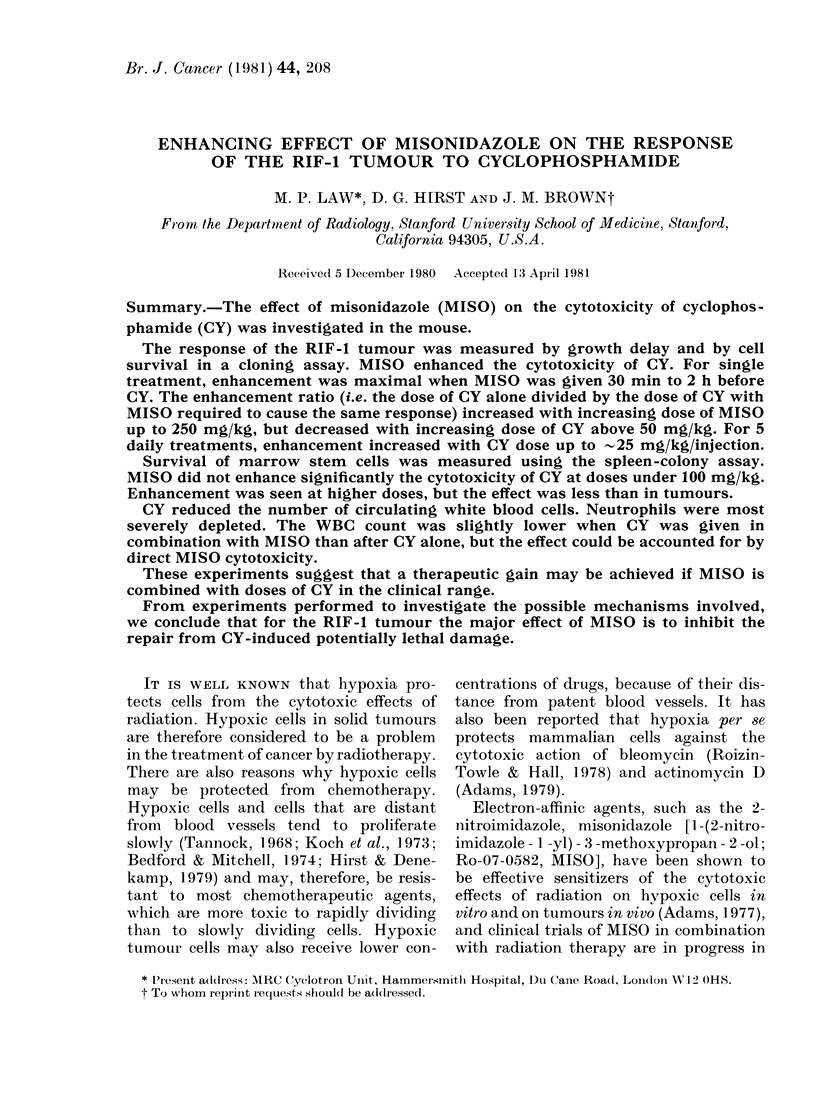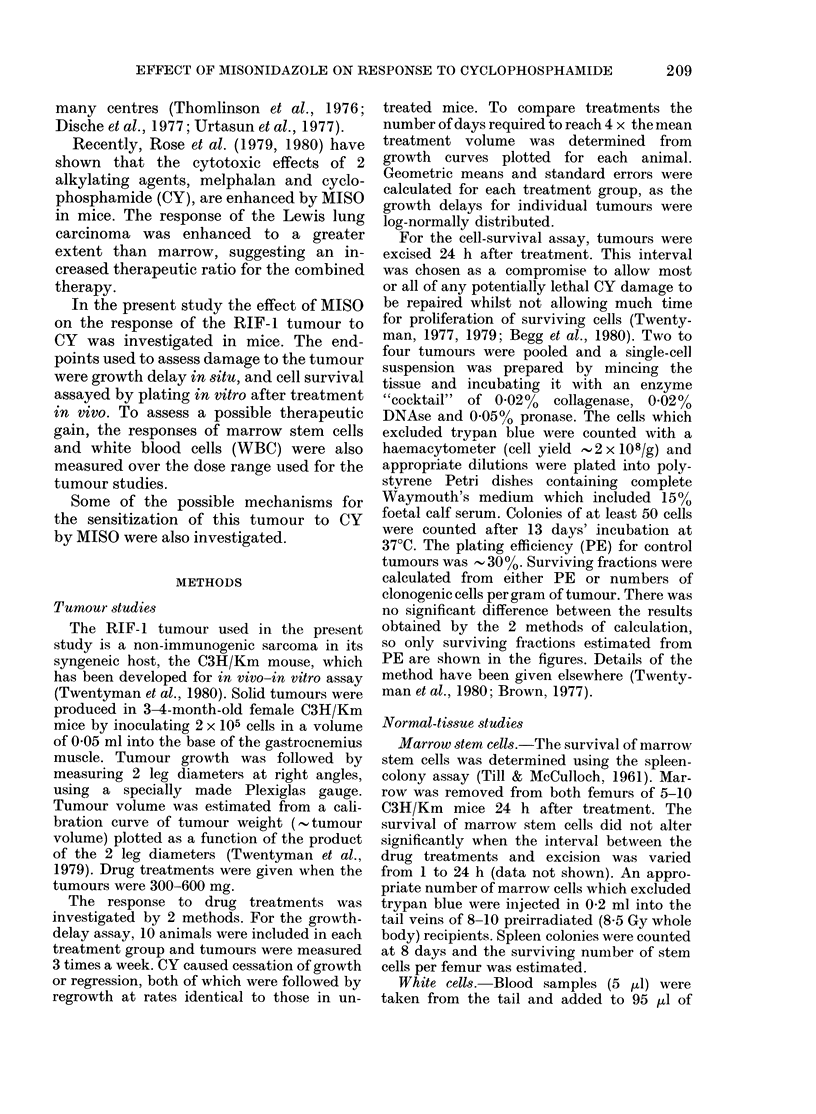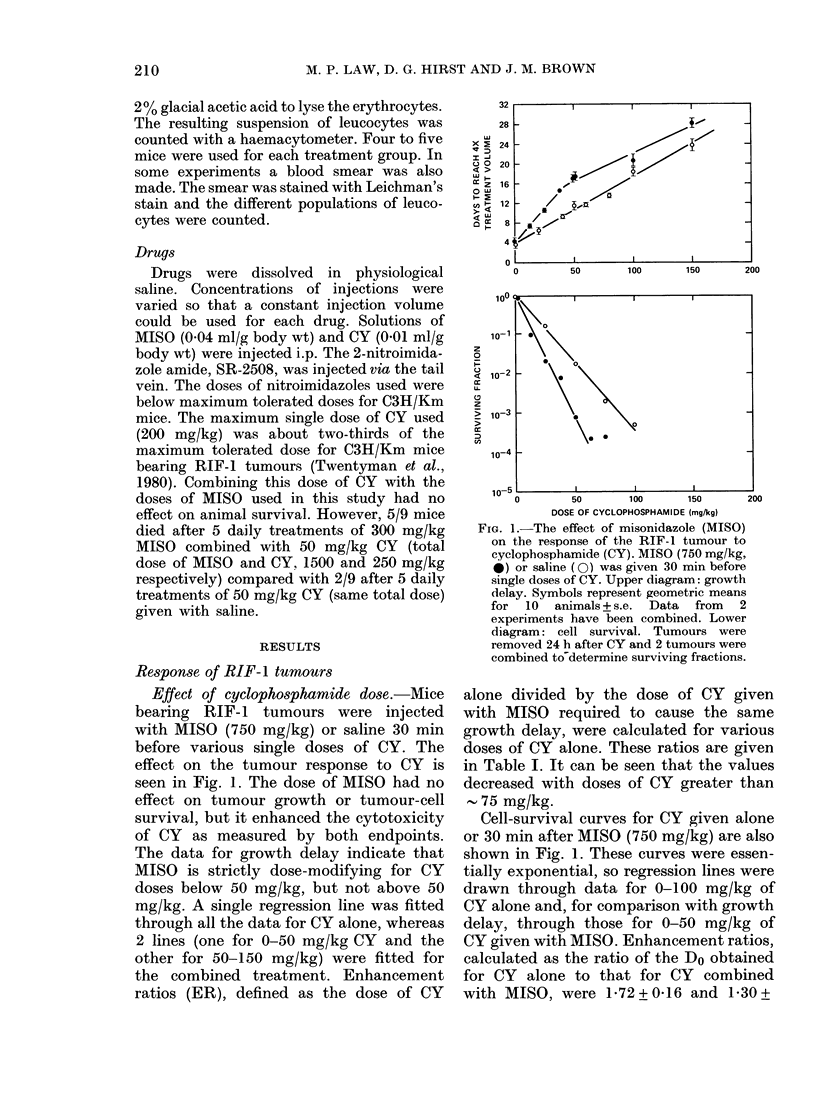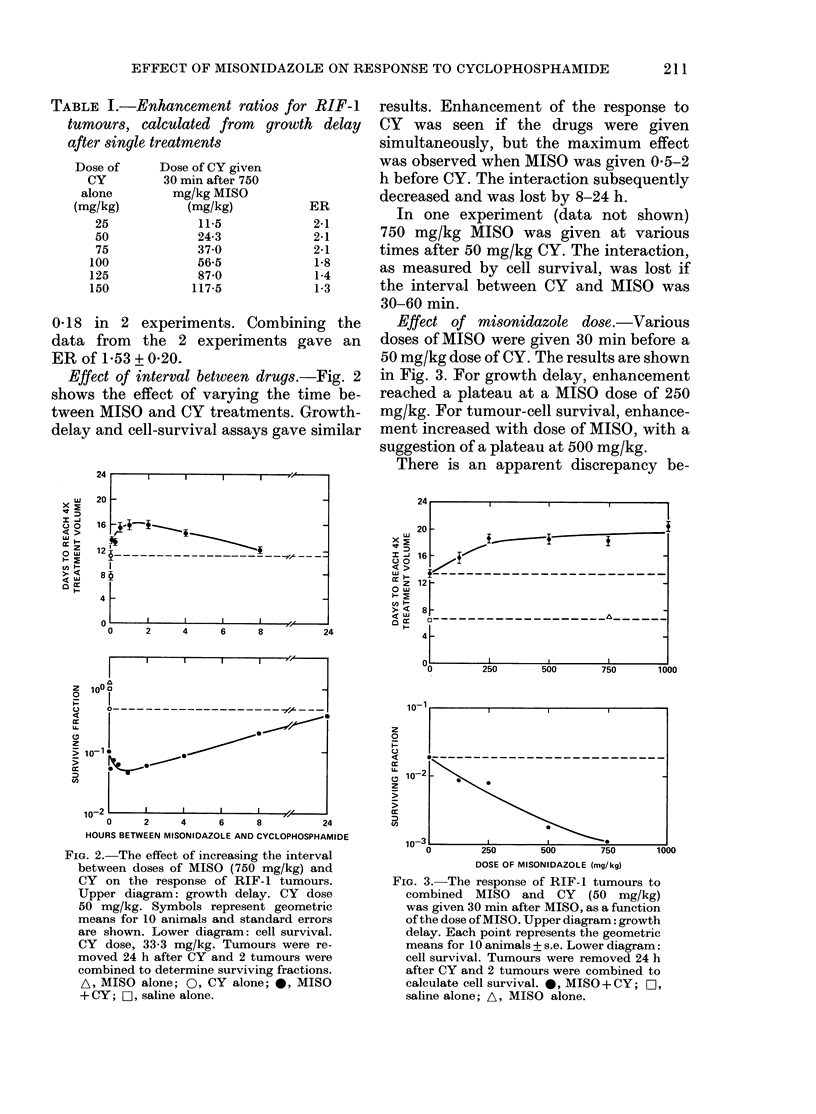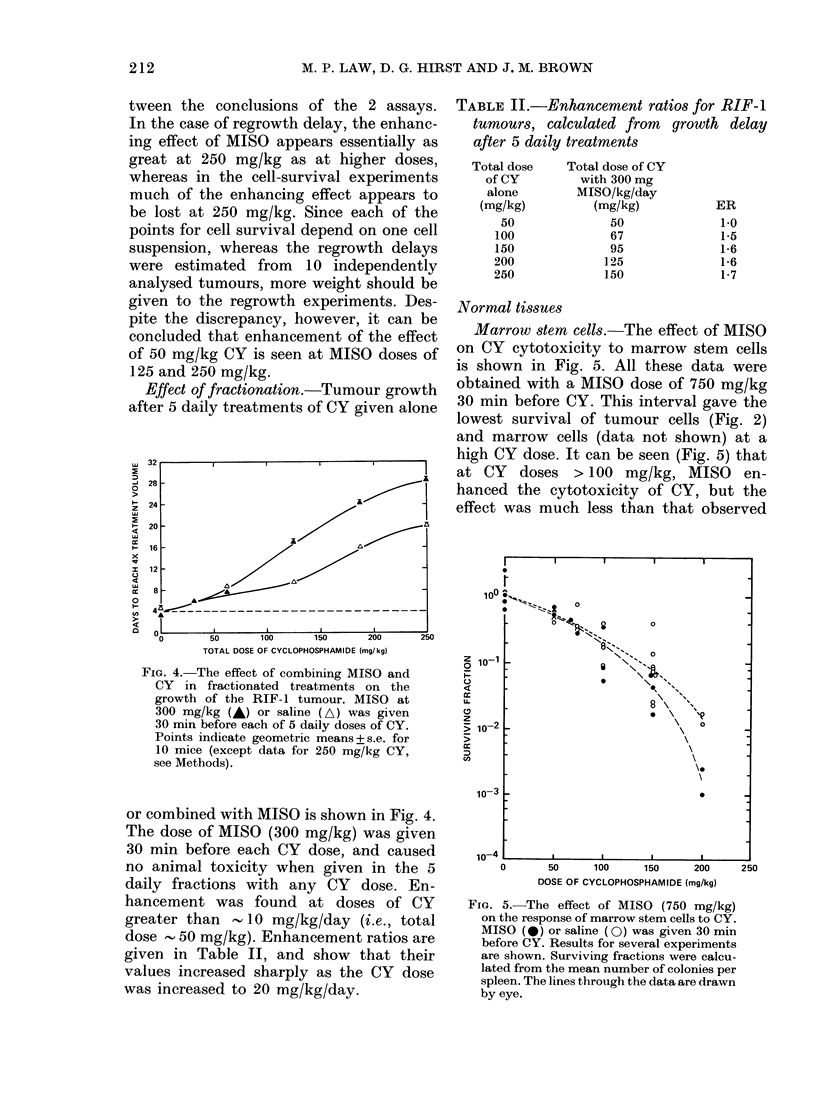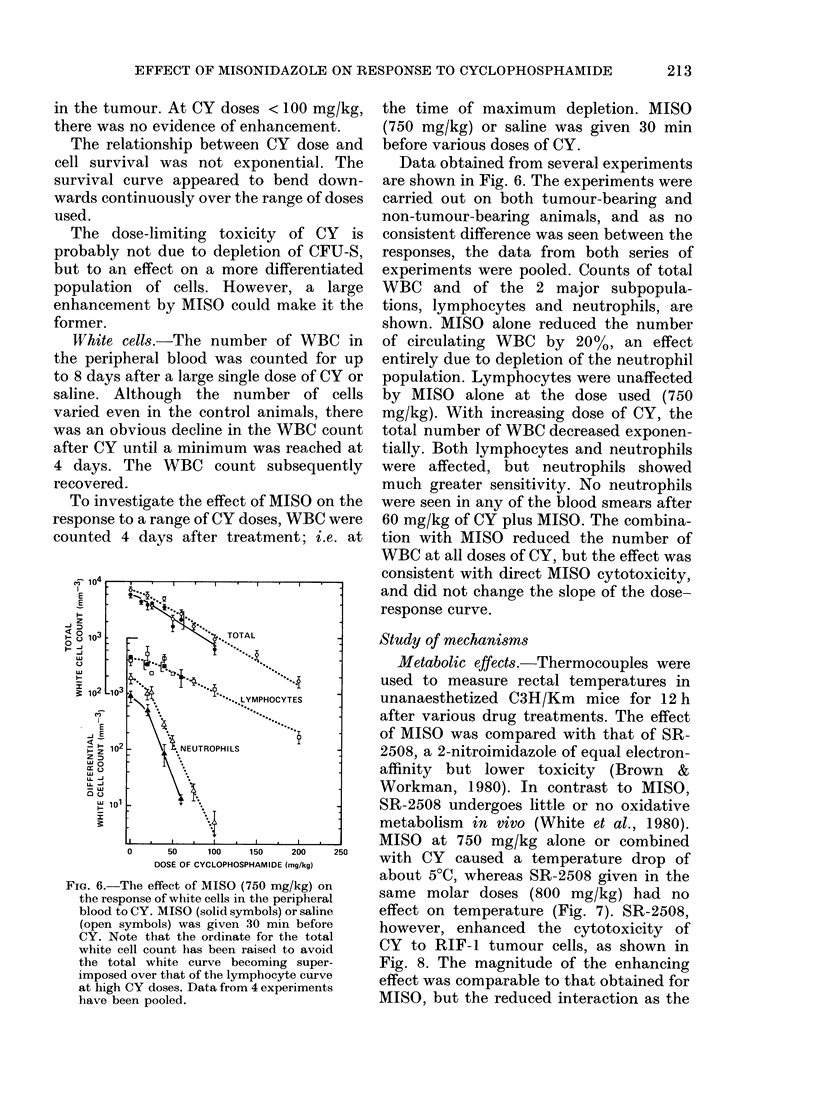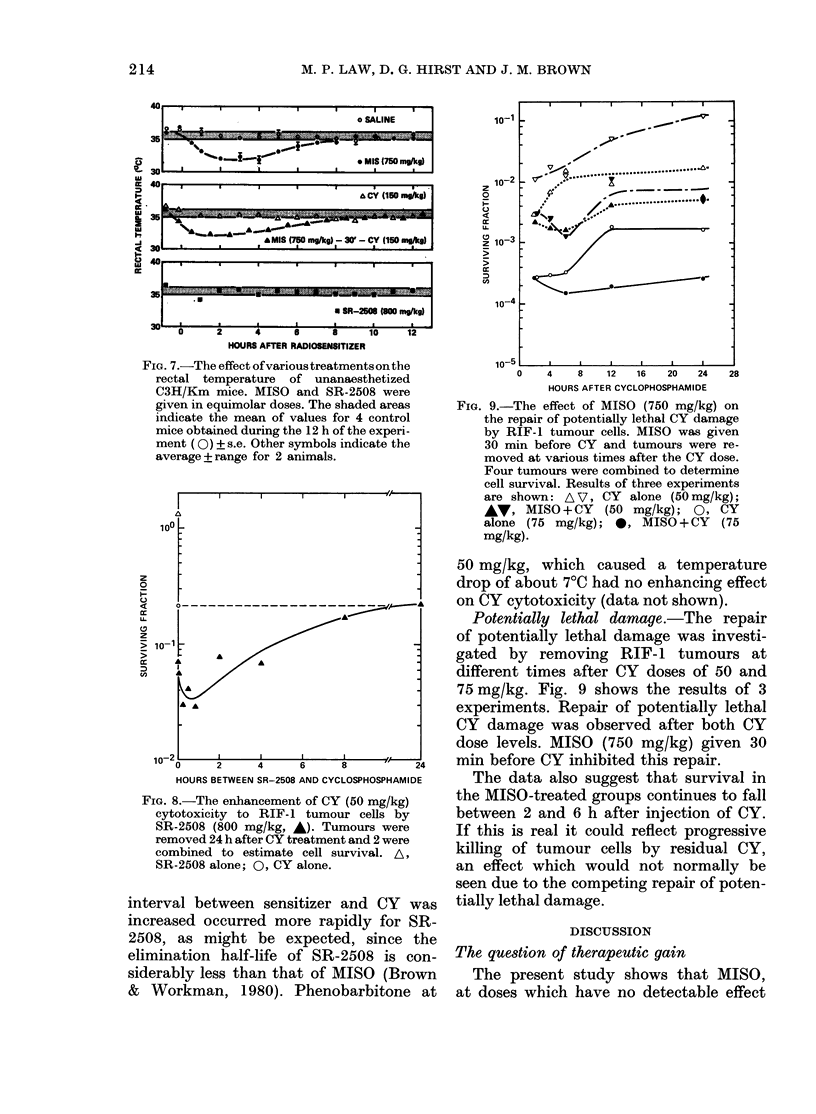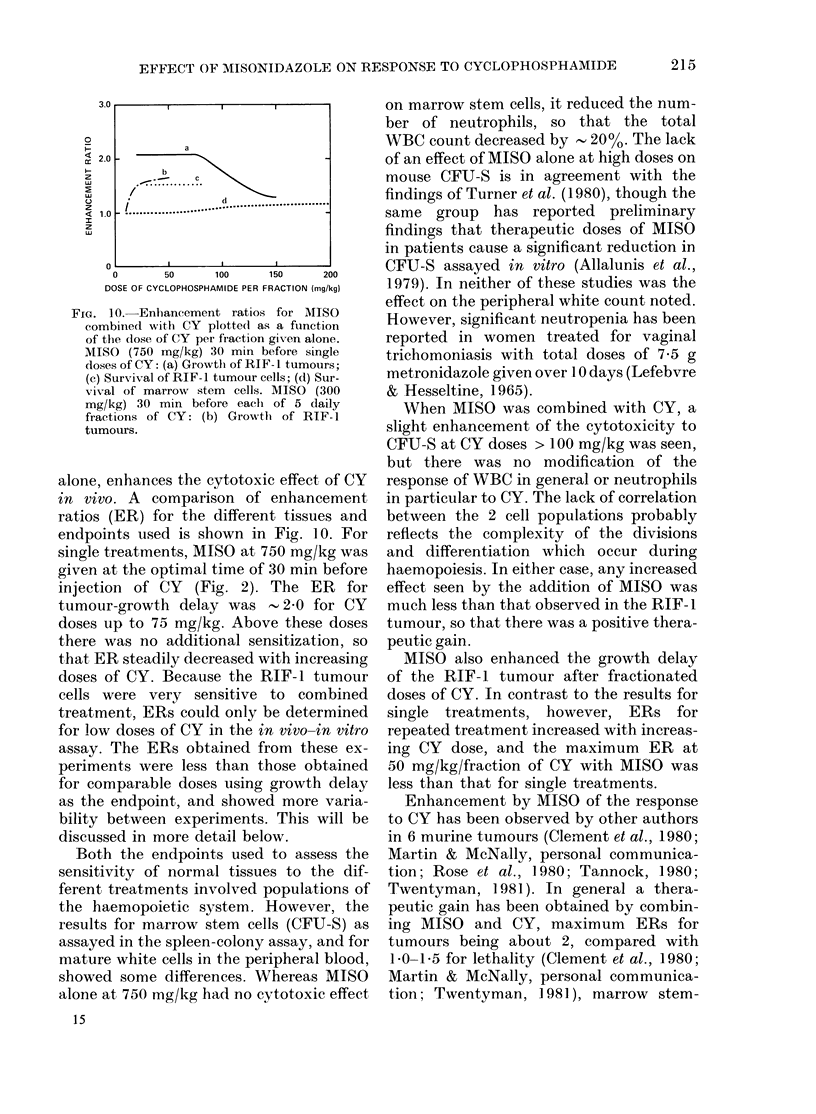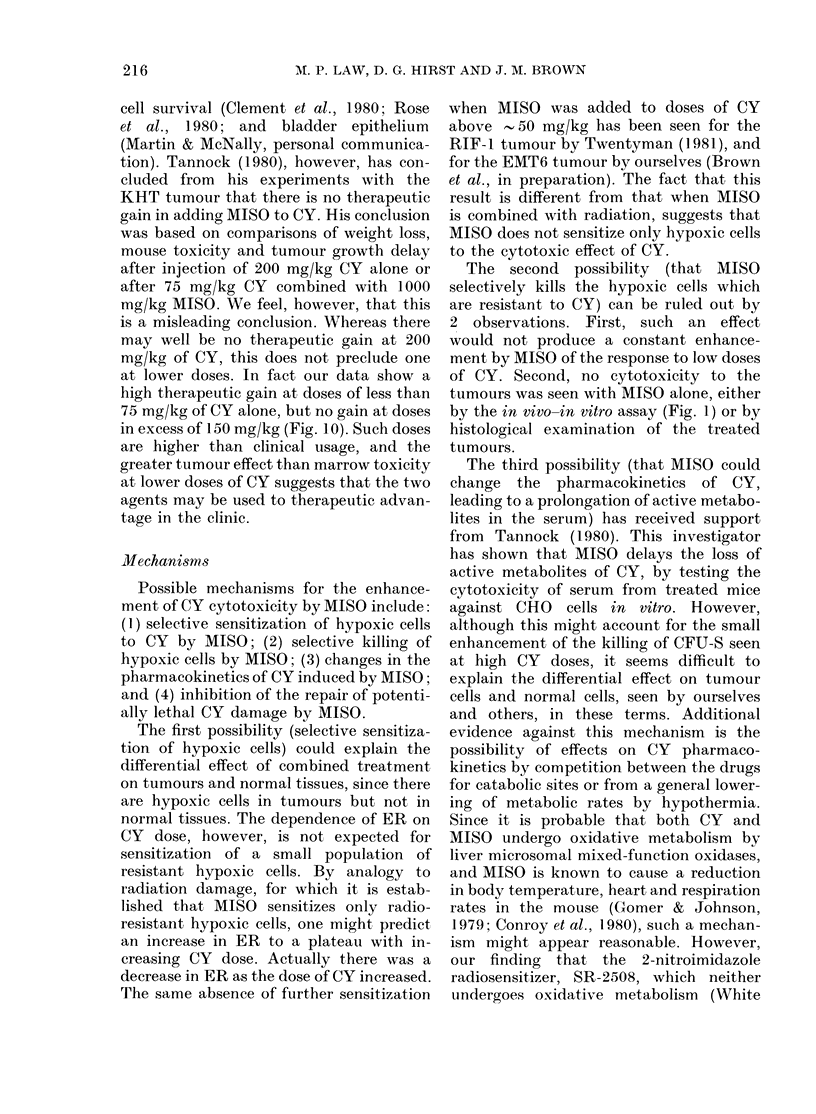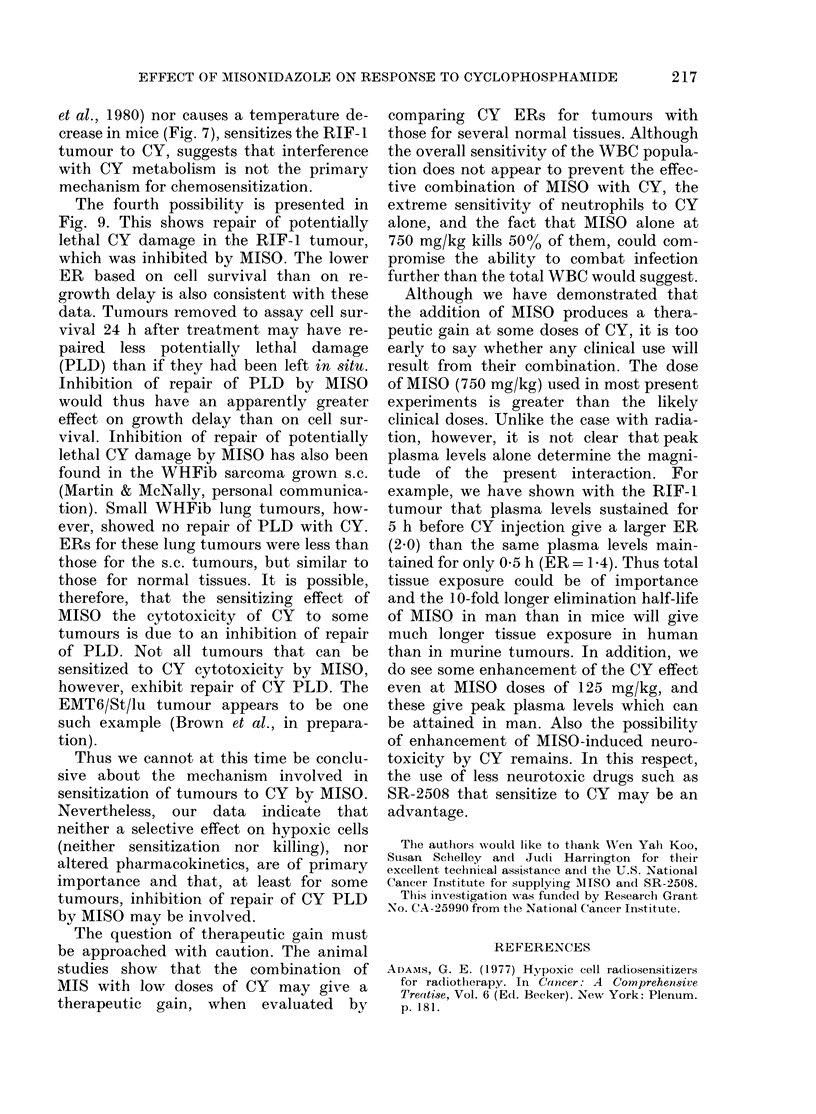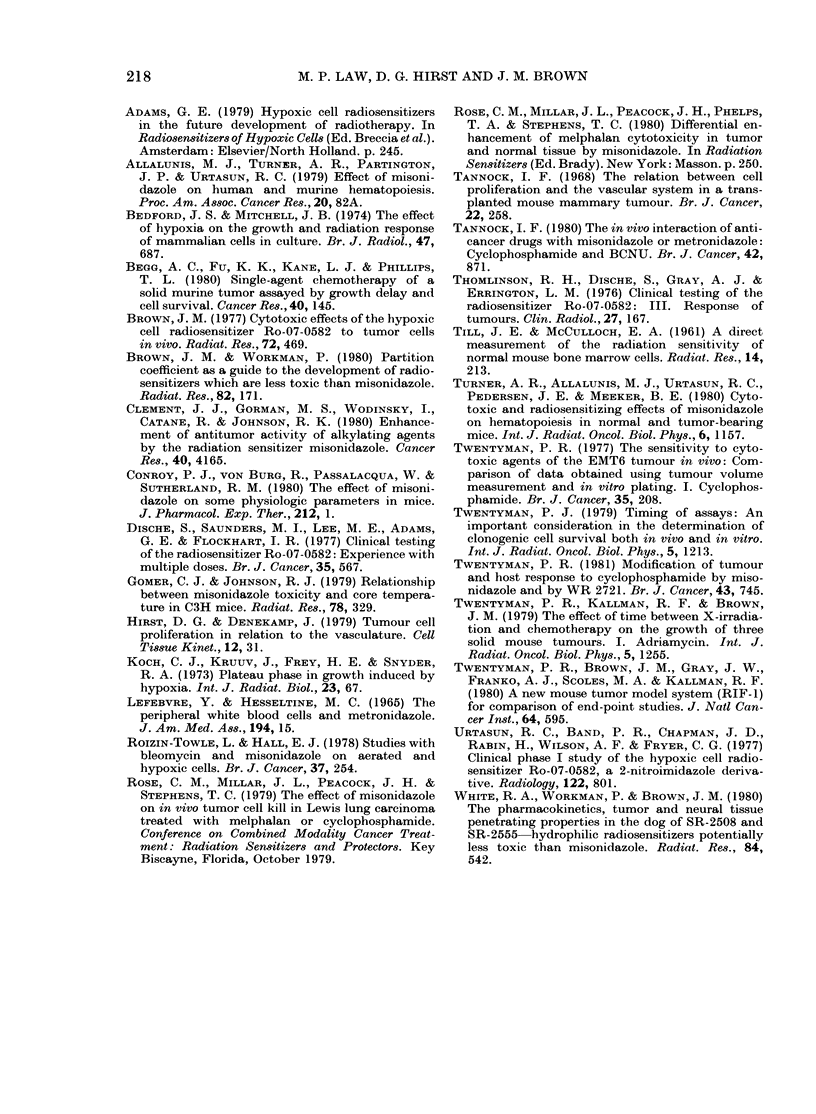# Enhancing effect of misonidazole on the response of the RIF-1 tumour to cyclophosphamide.

**DOI:** 10.1038/bjc.1981.172

**Published:** 1981-08

**Authors:** M. P. Law, D. G. Hirst, J. M. Brown

## Abstract

The effect of misonidazole (MISO) on the cytotoxicity of cyclophosphamide (CY) was investigated in the mouse. The response of the RIF-1 tumour was measured by growth delay and by cell survival in a cloning assay. MISO enhanced the cytotoxicity of CY. For single treatment, enhancement was maximal when MISO was given 30 min to 2 h before CY. The enhancement ratio (i.e. the dose of CY alone divided by the dose of CY with MISO required to cause the same response) increased with increasing dose of MISO up to 250 mg/kg, but decreased with increasing dose of CY above 50 mg/kg. For 5 daily treatments, enhancement increased with CY dose up to approximately 25 mg/kg/injection. Survival of marrow stem cells was measured using the spleen-colony assay. MISO did not enhance significantly the cytotoxicity of CY at doses under 100 mg/kg. Enhancement was seen at higher doses, but the effect was less than in tumours. CY reduced the number of circulating white blood cells. Neutrophils were most severely depleted. The WBC count was slightly lower when CY was given in combination with MISO than after CY alone, but the effect could be accounted for by direct MISO cytotoxicity. These experiences suggest that a therapeutic gain may be achieved if MISO is combined with doses of CY in the clinical range. From experiments performed to investigated the possible mechanisms involved, we conclude that for the RIF-1 tumour the major effect of MISO is to inhibit the repair from CY-induced potentially lethal damage.


*Br. J. Cancer* (1981) **44**, 208

### ENHANCING EFFECT OF MISONIDAZOLE ON THE RESPONSE OF THE RIF-1 TUMOUR TO CYCLOPHOSPHAMIDE

M. P. LAW*, D. G. HIRST AND J. M. BROWN†

*From the Department of Radiology, Stanford University School of Medicine, Stanford, California* 94305, *U.S.A.*

Received 5 December 1980 Accepted 13 April 1981

**Summary.—The effect of misonidazole (MISO) on the cytotoxicity of cyclophosphamide (CY) was investigated in the mouse.**

**The response of the RIF-1 tumour was measured by growth delay and by cell survival in a cloning assay. MISO enhanced the cytotoxicity of CY. For single treatment, enhancement was maximal when MISO was given 30 min to 2 h before CY. The enhancement ratio (*i.e.* the dose of CY alone divided by the dose of CY with MISO required to cause the same response) increased with increasing dose of MISO up to 250 mg/kg, but decreased with increasing dose of CY above 50 mg/kg. For 5 daily treatments, enhancement increased with CY dose up to ~25 mg/kg/injection.**

**Survival of marrow stem cells was measured using the spleen-colony assay. MISO did not enhance significantly the cytotoxicity of CY at doses under 100 mg/kg. Enhancement was seen at higher doses, but the effect was less than in tumours.**

**CY reduced the number of circulating white blood cells. Neutrophils were most severely depleted. The WBC count was slightly lower when CY was given in combination with MISO than after CY alone, but the effect could be accounted for by direct MISO cytotoxicity.**

**These experiments suggest that a therapeutic gain may be achieved if MISO is combined with doses of CY in the clinical range.**

**From experiments performed to investigate the possible mechanisms involved, we conclude that for the RIF-1 tumour the major effect of MISO is to inhibit the repair from CY-induced potentially lethal damage.**

It is well known that hypoxia protects cells from the cytotoxic effects of radiation. Hypoxic cells in solid tumours are therefore considered to be a problem in the treatment of cancer by radiotherapy. There are also reasons why hypoxic cells may be protected from chemotherapy. Hypoxic cells and cells that are distant from blood vessels tend to proliferate slowly (Tannock, 1968; Koch *et al.*, 1973; Bedford & Mitchell, 1974; Hirst & Denekamp, 1979) and may, therefore, be resistant to most chemotherapeutic agents, which are more toxic to rapidly dividing than to slowly dividing cells. Hypoxic tumour cells may also receive lower concentrations of drugs, because of their distance from patent blood vessels. It has also been reported that hypoxia *per se* protects mammalian cells against the cytotoxic action of bleomycin (Roizin-Towle & Hall, 1978) and actinomycin D (Adams, 1979).

Electron-affinic agents, such as the 2-nitroimidazole, misonidazole [1-(2-nitroimidazole-1-yl)-3-methoxypropan-2-ol; Ro-07-0582, MISO], have been shown to be effective sensitizers of the cytotoxic effects of radiation on hypoxic cells *in vitro* and on tumours *in vivo* (Adams, 1977), and clinical trials of MISO in combination with radiation therapy are in progress in

* Present address: MRC Cyclotron Unit, Hammersmith Hospital, Du Cane Road, London W12 0HS.

† To whom reprint requests should be addressed.

many centres (Thomlinson *et al.*, 1976; Dische *et al.*, 1977; Urtasun *et al.*, 1977).

Recently, Rose *et al.* (1979, 1980) have shown that the cytotoxic effects of 2 alkylating agents, melphalan and cyclophosphamide (CY), are enhanced by MISO in mice. The response of the Lewis lung carcinoma was enhanced to a greater extent than marrow, suggesting an increased therapeutic ratio for the combined therapy.

In the present study the effect of MISO on the response of the RIF-1 tumour to CY was investigated in mice. The endpoints used to assess damage to the tumour were growth delay *in situ*, and cell survival assayed by plating *in vitro* after treatment *in vivo*. To assess a possible therapeutic gain, the responses of marrow stem cells and white blood cells (WBC) were also measured over the dose range used for the tumour studies.

Some of the possible mechanisms for the sensitization of this tumour to CY by MISO were also investigated.

### METHODS

### *Tumour studies*

The RIF-1 tumour used in the present study is a non-immunogenic sarcoma in its syngeneic host, the C3H/Km mouse, which has been developed for *in vivo–in vitro* assay (Twentyman *et al.*, 1980). Solid tumours were produced in 3–4-month-old female C3H/Km mice by inoculating $2 \times 10^5$ cells in a volume of 0·05 ml into the base of the gastrocnemius muscle. Tumour growth was followed by measuring 2 leg diameters at right angles, using a specially made Plexiglas gauge. Tumour volume was estimated from a calibration curve of tumour weight ($\sim$tumour volume) plotted as a function of the product of the 2 leg diameters (Twentyman *et al.*, 1979). Drug treatments were given when the tumours were 300–600 mg.

The response to drug treatments was investigated by 2 methods. For the growth-delay assay, 10 animals were included in each treatment group and tumours were measured 3 times a week. CY caused cessation of growth or regression, both of which were followed by regrowth at rates identical to those in untreated mice. To compare treatments the number of days required to reach 4 × the mean treatment volume was determined from growth curves plotted for each animal. Geometric means and standard errors were calculated for each treatment group, as the growth delays for individual tumours were log-normally distributed.

For the cell-survival assay, tumours were excised 24 h after treatment. This interval was chosen as a compromise to allow most or all of any potentially lethal CY damage to be repaired whilst not allowing much time for proliferation of surviving cells (Twentyman, 1977, 1979; Begg *et al.*, 1980). Two to four tumours were pooled and a single-cell suspension was prepared by mincing the tissue and incubating it with an enzyme "cocktail" of 0·02% collagenase, 0·02% DNAse and 0·05% pronase. The cells which excluded trypan blue were counted with a haemacytometer (cell yield $\sim 2 \times 10^8$/g) and appropriate dilutions were plated into polystyrene Petri dishes containing complete Waymouth's medium which included 15% foetal calf serum. Colonies of at least 50 cells were counted after 13 days' incubation at 37°C. The plating efficiency (PE) for control tumours was $\sim$30%. Surviving fractions were calculated from either PE or numbers of clonogenic cells per gram of tumour. There was no significant difference between the results obtained by the 2 methods of calculation, so only surviving fractions estimated from PE are shown in the figures. Details of the method have been given elsewhere (Twentyman *et al.*, 1980; Brown, 1977).

### *Normal-tissue studies*

*Marrow stem cells.*—The survival of marrow stem cells was determined using the spleen-colony assay (Till & McCulloch, 1961). Marrow was removed from both femurs of 5–10 C3H/Km mice 24 h after treatment. The survival of marrow stem cells did not alter significantly when the interval between the drug treatments and excision was varied from 1 to 24 h (data not shown). An appropriate number of marrow cells which excluded trypan blue were injected in 0·2 ml into the tail veins of 8–10 preirradiated (8·5 Gy whole body) recipients. Spleen colonies were counted at 8 days and the surviving number of stem cells per femur was estimated.

*White cells.*—Blood samples (5 $\mu$l) were taken from the tail and added to 95 $\mu$l of

2% glacial acetic acid to lyse the erythrocytes. The resulting suspension of leucocytes was counted with a haemacytometer. Four to five mice were used for each treatment group. In some experiments a blood smear was also made. The smear was stained with Leichman's stain and the different populations of leucocytes were counted.

*Drugs*

Drugs were dissolved in physiological saline. Concentrations of injections were varied so that a constant injection volume could be used for each drug. Solutions of MISO (0·04 ml/g body wt) and CY (0·01 ml/g body wt) were injected i.p. The 2-nitroimidazole amide, SR-2508, was injected *via* the tail vein. The doses of nitroimidazoles used were below maximum tolerated doses for C3H/Km mice. The maximum single dose of CY used (200 mg/kg) was about two-thirds of the maximum tolerated dose for C3H/Km mice bearing RIF-1 tumours (Twentyman *et al.*, 1980). Combining this dose of CY with the doses of MISO used in this study had no effect on animal survival. However, 5/9 mice died after 5 daily treatments of 300 mg/kg MISO combined with 50 mg/kg CY (total dose of MISO and CY, 1500 and 250 mg/kg respectively) compared with 2/9 after 5 daily treatments of 50 mg/kg CY (same total dose) given with saline.

FIG. 1.—The effect of misonidazole (MISO) on the response of the RIF-1 tumour to cyclophosphamide (CY). MISO (750 mg/kg, ●) or saline (○) was given 30 min before single doses of CY. Upper diagram: growth delay. Symbols represent geometric means for 10 animals ± s.e. Data from 2 experiments have been combined. Lower diagram: cell survival. Tumours were removed 24 h after CY and 2 tumours were combined to determine surviving fractions.

### RESULTS

*Response of RIF-1 tumours*

*Effect of cyclophosphamide dose.*—Mice bearing RIF-1 tumours were injected with MISO (750 mg/kg) or saline 30 min before various single doses of CY. The effect on the tumour response to CY is seen in Fig. 1. The dose of MISO had no effect on tumour growth or tumour-cell survival, but it enhanced the cytotoxicity of CY as measured by both endpoints. The data for growth delay indicate that MISO is strictly dose-modifying for CY doses below 50 mg/kg, but not above 50 mg/kg. A single regression line was fitted through all the data for CY alone, whereas 2 lines (one for 0–50 mg/kg CY and the other for 50–150 mg/kg) were fitted for the combined treatment. Enhancement ratios (ER), defined as the dose of CY alone divided by the dose of CY given with MISO required to cause the same growth delay, were calculated for various doses of CY alone. These ratios are given in Table I. It can be seen that the values decreased with doses of CY greater than ~75 mg/kg.

Cell-survival curves for CY given alone or 30 min after MISO (750 mg/kg) are also shown in Fig. 1. These curves were essentially exponential, so regression lines were drawn through data for 0–100 mg/kg of CY alone and, for comparison with growth delay, through those for 0–50 mg/kg of CY given with MISO. Enhancement ratios, calculated as the ratio of the $D_0$ obtained for CY alone to that for CY combined with MISO, were 1·72 ± 0·16 and 1·30 ±

TABLE I.—*Enhancement ratios for RIF-1 tumours, calculated from growth delay after single treatments*

| Dose of CY alone (mg/kg) | Dose of CY given 30 min after 750 mg/kg MISO (mg/kg) | ER |
|---|---|---|
| 25 | 11·5 | 2·1 |
| 50 | 24·3 | 2·1 |
| 75 | 37·0 | 2·1 |
| 100 | 56·5 | 1·8 |
| 125 | 87·0 | 1·4 |
| 150 | 117·5 | 1·3 |

0·18 in 2 experiments. Combining the data from the 2 experiments gave an ER of 1·53 ± 0·20.

*Effect of interval between drugs.*—Fig. 2 shows the effect of varying the time between MISO and CY treatments. Growth-delay and cell-survival assays gave similar results. Enhancement of the response to CY was seen if the drugs were given simultaneously, but the maximum effect was observed when MISO was given 0·5–2 h before CY. The interaction subsequently decreased and was lost by 8–24 h.

In one experiment (data not shown) 750 mg/kg MISO was given at various times after 50 mg/kg CY. The interaction, as measured by cell survival, was lost if the interval between CY and MISO was 30–60 min.

*Effect of misonidazole dose.*—Various doses of MISO were given 30 min before a 50 mg/kg dose of CY. The results are shown in Fig. 3. For growth delay, enhancement reached a plateau at a MISO dose of 250 mg/kg. For tumour-cell survival, enhancement increased with dose of MISO, with a suggestion of a plateau at 500 mg/kg.

There is an apparent discrepancy be-

FIG. 2.—The effect of increasing the interval between doses of MISO (750 mg/kg) and CY on the response of RIF-1 tumours. Upper diagram: growth delay. CY dose 50 mg/kg. Symbols represent geometric means for 10 animals and standard errors are shown. Lower diagram: cell survival. CY dose, 33·3 mg/kg. Tumours were removed 24 h after CY and 2 tumours were combined to determine surviving fractions. △, MISO alone; ○, CY alone; ●, MISO + CY; □, saline alone.

FIG. 3.—The response of RIF-1 tumours to combined MISO and CY (50 mg/kg) was given 30 min after MISO, as a function of the dose of MISO. Upper diagram: growth delay. Each point represents the geometric means for 10 animals ± s.e. Lower diagram: cell survival. Tumours were removed 24 h after CY and 2 tumours were combined to calculate cell survival. ●, MISO + CY; □, saline alone; △, MISO alone.

tween the conclusions of the 2 assays. In the case of regrowth delay, the enhancing effect of MISO appears essentially as great at 250 mg/kg as at higher doses, whereas in the cell-survival experiments much of the enhancing effect appears to be lost at 250 mg/kg. Since each of the points for cell survival depend on one cell suspension, whereas the regrowth delays were estimated from 10 independently analysed tumours, more weight should be given to the regrowth experiments. Despite the discrepancy, however, it can be concluded that enhancement of the effect of 50 mg/kg CY is seen at MISO doses of 125 and 250 mg/kg.

*Effect of fractionation.*—Tumour growth after 5 daily treatments of CY given alone

FIG. 4.—The effect of combining MISO and CY in fractionated treatments on the growth of the RIF-1 tumour. MISO at 300 mg/kg (▲) or saline (△) was given 30 min before each of 5 daily doses of CY. Points indicate geometric means ± s.e. for 10 mice (except data for 250 mg/kg CY, see Methods).

or combined with MISO is shown in Fig. 4. The dose of MISO (300 mg/kg) was given 30 min before each CY dose, and caused no animal toxicity when given in the 5 daily fractions with any CY dose. Enhancement was found at doses of CY greater than ~10 mg/kg/day (*i.e.*, total dose ~50 mg/kg). Enhancement ratios are given in Table II, and show that their values increased sharply as the CY dose was increased to 20 mg/kg/day.

TABLE II.—*Enhancement ratios for RIF-1 tumours, calculated from growth delay after 5 daily treatments*

| Total dose of CY alone (mg/kg) | Total dose of CY with 300 mg MISO/kg/day (mg/kg) | ER |
|---|---|---|
| 50 | 50 | 1·0 |
| 100 | 67 | 1·5 |
| 150 | 95 | 1·6 |
| 200 | 125 | 1·6 |
| 250 | 150 | 1·7 |

*Normal tissues*

*Marrow stem cells.*—The effect of MISO on CY cytotoxicity to marrow stem cells is shown in Fig. 5. All these data were obtained with a MISO dose of 750 mg/kg 30 min before CY. This interval gave the lowest survival of tumour cells (Fig. 2) and marrow cells (data not shown) at a high CY dose. It can be seen (Fig. 5) that at CY doses >100 mg/kg, MISO enhanced the cytotoxicity of CY, but the effect was much less than that observed

FIG. 5.—The effect of MISO (750 mg/kg) on the response of marrow stem cells to CY. MISO (●) or saline (○) was given 30 min before CY. Results for several experiments are shown. Surviving fractions were calculated from the mean number of colonies per spleen. The lines through the data are drawn by eye.

in the tumour. At CY doses < 100 mg/kg, there was no evidence of enhancement.

The relationship between CY dose and cell survival was not exponential. The survival curve appeared to bend downwards continuously over the range of doses used.

The dose-limiting toxicity of CY is probably not due to depletion of CFU-S, but to an effect on a more differentiated population of cells. However, a large enhancement by MISO could make it the former.

*White cells.*—The number of WBC in the peripheral blood was counted for up to 8 days after a large single dose of CY or saline. Although the number of cells varied even in the control animals, there was an obvious decline in the WBC count after CY until a minimum was reached at 4 days. The WBC count subsequently recovered.

To investigate the effect of MISO on the response to a range of CY doses, WBC were counted 4 days after treatment; *i.e.* at the time of maximum depletion. MISO (750 mg/kg) or saline was given 30 min before various doses of CY.

FIG. 6.—The effect of MISO (750 mg/kg) on the response of white cells in the peripheral blood to CY. MISO (solid symbols) or saline (open symbols) was given 30 min before CY. Note that the ordinate for the total white cell count has been raised to avoid the total white curve becoming superimposed over that of the lymphocyte curve at high CY doses. Data from 4 experiments have been pooled.

Data obtained from several experiments are shown in Fig. 6. The experiments were carried out on both tumour-bearing and non-tumour-bearing animals, and as no consistent difference was seen between the responses, the data from both series of experiments were pooled. Counts of total WBC and of the 2 major subpopulations, lymphocytes and neutrophils, are shown. MISO alone reduced the number of circulating WBC by 20%, an effect entirely due to depletion of the neutrophil population. Lymphocytes were unaffected by MISO alone at the dose used (750 mg/kg). With increasing dose of CY, the total number of WBC decreased exponentially. Both lymphocytes and neutrophils were affected, but neutrophils showed much greater sensitivity. No neutrophils were seen in any of the blood smears after 60 mg/kg of CY plus MISO. The combination with MISO reduced the number of WBC at all doses of CY, but the effect was consistent with direct MISO cytotoxicity, and did not change the slope of the dose–response curve.

### *Study of mechanisms*

*Metabolic effects.*—Thermocouples were used to measure rectal temperatures in unanaesthetized C3H/Km mice for 12 h after various drug treatments. The effect of MISO was compared with that of SR-2508, a 2-nitroimidazole of equal electron-affinity but lower toxicity (Brown & Workman, 1980). In contrast to MISO, SR-2508 undergoes little or no oxidative metabolism *in vivo* (White *et al.*, 1980). MISO at 750 mg/kg alone or combined with CY caused a temperature drop of about 5°C, whereas SR-2508 given in the same molar doses (800 mg/kg) had no effect on temperature (Fig. 7). SR-2508, however, enhanced the cytotoxicity of CY to RIF-1 tumour cells, as shown in Fig. 8. The magnitude of the enhancing effect was comparable to that obtained for MISO, but the reduced interaction as the

FIG. 7.—The effect of various treatments on the rectal temperature of unanaesthetized C3H/Km mice. MISO and SR-2508 were given in equimolar doses. The shaded areas indicate the mean of values for 4 control mice obtained during the 12 h of the experiment (○) ± s.e. Other symbols indicate the average ± range for 2 animals.

FIG. 8.—The enhancement of CY (50 mg/kg) cytotoxicity to RIF-1 tumour cells by SR-2508 (800 mg/kg, ▲). Tumours were removed 24 h after CY treatment and 2 were combined to estimate cell survival. △, SR-2508 alone; ○, CY alone.

FIG. 9.—The effect of MISO (750 mg/kg) on the repair of potentially lethal CY damage by RIF-1 tumour cells. MISO was given 30 min before CY and tumours were removed at various times after the CY dose. Four tumours were combined to determine cell survival. Results of three experiments are shown: △▽, CY alone (50 mg/kg); ▲▼, MISO + CY (50 mg/kg); ○, CY alone (75 mg/kg); ●, MISO + CY (75 mg/kg).

interval between sensitizer and CY was increased occurred more rapidly for SR-2508, as might be expected, since the elimination half-life of SR-2508 is considerably less than that of MISO (Brown & Workman, 1980). Phenobarbitone at 50 mg/kg, which caused a temperature drop of about 7°C had no enhancing effect on CY cytotoxicity (data not shown).

*Potentially lethal damage.*—The repair of potentially lethal damage was investigated by removing RIF-1 tumours at different times after CY doses of 50 and 75 mg/kg. Fig. 9 shows the results of 3 experiments. Repair of potentially lethal CY damage was observed after both CY dose levels. MISO (750 mg/kg) given 30 min before CY inhibited this repair.

The data also suggest that survival in the MISO-treated groups continues to fall between 2 and 6 h after injection of CY. If this is real it could reflect progressive killing of tumour cells by residual CY, an effect which would not normally be seen due to the competing repair of potentially lethal damage.

### DISCUSSION

*The question of therapeutic gain*

The present study shows that MISO, at doses which have no detectable effect

FIG. 10.—Enhancement ratios for MISO combined with CY plotted as a function of the dose of CY per fraction given alone. MISO (750 mg/kg) 30 min before single doses of CY: (a) Growth of RIF-1 tumours; (c) Survival of RIF-1 tumour cells; (d) Survival of marrow stem cells. MISO (300 mg/kg) 30 min before each of 5 daily fractions of CY: (b) Growth of RIF-1 tumours.

alone, enhances the cytotoxic effect of CY *in vivo*. A comparison of enhancement ratios (ER) for the different tissues and endpoints used is shown in Fig. 10. For single treatments, MISO at 750 mg/kg was given at the optimal time of 30 min before injection of CY (Fig. 2). The ER for tumour-growth delay was ~2·0 for CY doses up to 75 mg/kg. Above these doses there was no additional sensitization, so that ER steadily decreased with increasing doses of CY. Because the RIF-1 tumour cells were very sensitive to combined treatment, ERs could only be determined for low doses of CY in the *in vivo–in vitro* assay. The ERs obtained from these experiments were less than those obtained for comparable doses using growth delay as the endpoint, and showed more variability between experiments. This will be discussed in more detail below.

Both the endpoints used to assess the sensitivity of normal tissues to the different treatments involved populations of the haemopoietic system. However, the results for marrow stem cells (CFU-S) as assayed in the spleen-colony assay, and for mature white cells in the peripheral blood, showed some differences. Whereas MISO alone at 750 mg/kg had no cytotoxic effect on marrow stem cells, it reduced the number of neutrophils, so that the total WBC count decreased by ~20%. The lack of an effect of MISO alone at high doses on mouse CFU-S is in agreement with the findings of Turner *et al.* (1980), though the same group has reported preliminary findings that therapeutic doses of MISO in patients cause a significant reduction in CFU-S assayed *in vitro* (Allalunis *et al.*, 1979). In neither of these studies was the effect on the peripheral white count noted. However, significant neutropenia has been reported in women treated for vaginal trichomoniasis with total doses of 7·5 g metronidazole given over 10 days (Lefebvre & Hesseltine, 1965).

When MISO was combined with CY, a slight enhancement of the cytotoxicity to CFU-S at CY doses > 100 mg/kg was seen, but there was no modification of the response of WBC in general or neutrophils in particular to CY. The lack of correlation between the 2 cell populations probably reflects the complexity of the divisions and differentiation which occur during haemopoiesis. In either case, any increased effect seen by the addition of MISO was much less than that observed in the RIF-1 tumour, so that there was a positive therapeutic gain.

MISO also enhanced the growth delay of the RIF-1 tumour after fractionated doses of CY. In contrast to the results for single treatments, however, ERs for repeated treatment increased with increasing CY dose, and the maximum ER at 50 mg/kg/fraction of CY with MISO was less than that for single treatments.

Enhancement by MISO of the response to CY has been observed by other authors in 6 murine tumours (Clement *et al.*, 1980; Martin & McNally, personal communication; Rose *et al.*, 1980; Tannock, 1980; Twentyman, 1981). In general a therapeutic gain has been obtained by combining MISO and CY, maximum ERs for tumours being about 2, compared with 1·0–1·5 for lethality (Clement *et al.*, 1980; Martin & McNally, personal communication; Twentyman, 1981), marrow stemcell survival (Clement *et al.*, 1980; Rose *et al.*, 1980; and bladder epithelium (Martin & McNally, personal communication). Tannock (1980), however, has concluded from his experiments with the KHT tumour that there is no therapeutic gain in adding MISO to CY. His conclusion was based on comparisons of weight loss, mouse toxicity and tumour growth delay after injection of 200 mg/kg CY alone or after 75 mg/kg CY combined with 1000 mg/kg MISO. We feel, however, that this is a misleading conclusion. Whereas there may well be no therapeutic gain at 200 mg/kg of CY, this does not preclude one at lower doses. In fact our data show a high therapeutic gain at doses of less than 75 mg/kg of CY alone, but no gain at doses in excess of 150 mg/kg (Fig. 10). Such doses are higher than clinical usage, and the greater tumour effect than marrow toxicity at lower doses of CY suggests that the two agents may be used to therapeutic advantage in the clinic.

*Mechanisms*

Possible mechanisms for the enhancement of CY cytotoxicity by MISO include: (1) selective sensitization of hypoxic cells to CY by MISO; (2) selective killing of hypoxic cells by MISO; (3) changes in the pharmacokinetics of CY induced by MISO; and (4) inhibition of the repair of potentially lethal CY damage by MISO.

The first possibility (selective sensitization of hypoxic cells) could explain the differential effect of combined treatment on tumours and normal tissues, since there are hypoxic cells in tumours but not in normal tissues. The dependence of ER on CY dose, however, is not expected for sensitization of a small population of resistant hypoxic cells. By analogy to radiation damage, for which it is established that MISO sensitizes only radioresistant hypoxic cells, one might predict an increase in ER to a plateau with increasing CY dose. Actually there was a decrease in ER as the dose of CY increased. The same absence of further sensitization when MISO was added to doses of CY above $\sim 50$ mg/kg has been seen for the RIF-1 tumour by Twentyman (1981), and for the EMT6 tumour by ourselves (Brown *et al.*, in preparation). The fact that this result is different from that when MISO is combined with radiation, suggests that MISO does not sensitize only hypoxic cells to the cytotoxic effect of CY.

The second possibility (that MISO selectively kills the hypoxic cells which are resistant to CY) can be ruled out by 2 observations. First, such an effect would not produce a constant enhancement by MISO of the response to low doses of CY. Second, no cytotoxicity to the tumours was seen with MISO alone, either by the *in vivo–in vitro* assay (Fig. 1) or by histological examination of the treated tumours.

The third possibility (that MISO could change the pharmacokinetics of CY, leading to a prolongation of active metabolites in the serum) has received support from Tannock (1980). This investigator has shown that MISO delays the loss of active metabolites of CY, by testing the cytotoxicity of serum from treated mice against CHO cells *in vitro*. However, although this might account for the small enhancement of the killing of CFU-S seen at high CY doses, it seems difficult to explain the differential effect on tumour cells and normal cells, seen by ourselves and others, in these terms. Additional evidence against this mechanism is the possibility of effects on CY pharmacokinetics by competition between the drugs for catabolic sites or from a general lowering of metabolic rates by hypothermia. Since it is probable that both CY and MISO undergo oxidative metabolism by liver microsomal mixed-function oxidases, and MISO is known to cause a reduction in body temperature, heart and respiration rates in the mouse (Gomer & Johnson, 1979; Conroy *et al.*, 1980), such a mechanism might appear reasonable. However, our finding that the 2-nitroimidazole radiosensitizer, SR-2508, which neither undergoes oxidative metabolism (White

*et al.*, 1980) nor causes a temperature decrease in mice (Fig. 7), sensitizes the RIF-1 tumour to CY, suggests that interference with CY metabolism is not the primary mechanism for chemosensitization.

The fourth possibility is presented in Fig. 9. This shows repair of potentially lethal CY damage in the RIF-1 tumour, which was inhibited by MISO. The lower ER based on cell survival than on regrowth delay is also consistent with these data. Tumours removed to assay cell survival 24 h after treatment may have repaired less potentially lethal damage (PLD) than if they had been left *in situ*. Inhibition of repair of PLD by MISO would thus have an apparently greater effect on growth delay than on cell survival. Inhibition of repair of potentially lethal CY damage by MISO has also been found in the WHFib sarcoma grown s.c. (Martin & McNally, personal communication). Small WHFib lung tumours, however, showed no repair of PLD with CY. ERs for these lung tumours were less than those for the s.c. tumours, but similar to those for normal tissues. It is possible, therefore, that the sensitizing effect of MISO the cytotoxicity of CY to some tumours is due to an inhibition of repair of PLD. Not all tumours that can be sensitized to CY cytotoxicity by MISO, however, exhibit repair of CY PLD. The EMT6/St/lu tumour appears to be one such example (Brown *et al.*, in preparation).

Thus we cannot at this time be conclusive about the mechanism involved in sensitization of tumours to CY by MISO. Nevertheless, our data indicate that neither a selective effect on hypoxic cells (neither sensitization nor killing), nor altered pharmacokinetics, are of primary importance and that, at least for some tumours, inhibition of repair of CY PLD by MISO may be involved.

The question of therapeutic gain must be approached with caution. The animal studies show that the combination of MIS with low doses of CY may give a therapeutic gain, when evaluated by comparing CY ERs for tumours with those for several normal tissues. Although the overall sensitivity of the WBC population does not appear to prevent the effective combination of MISO with CY, the extreme sensitivity of neutrophils to CY alone, and the fact that MISO alone at 750 mg/kg kills 50% of them, could compromise the ability to combat infection further than the total WBC would suggest.

Although we have demonstrated that the addition of MISO produces a therapeutic gain at some doses of CY, it is too early to say whether any clinical use will result from their combination. The dose of MISO (750 mg/kg) used in most present experiments is greater than the likely clinical doses. Unlike the case with radiation, however, it is not clear that peak plasma levels alone determine the magnitude of the present interaction. For example, we have shown with the RIF-1 tumour that plasma levels sustained for 5 h before CY injection give a larger ER (2·0) than the same plasma levels maintained for only 0·5 h (ER = 1·4). Thus total tissue exposure could be of importance and the 10-fold longer elimination half-life of MISO in man than in mice will give much longer tissue exposure in human than in murine tumours. In addition, we do see some enhancement of the CY effect even at MISO doses of 125 mg/kg, and these give peak plasma levels which can be attained in man. Also the possibility of enhancement of MISO-induced neurotoxicity by CY remains. In this respect, the use of less neurotoxic drugs such as SR-2508 that sensitize to CY may be an advantage.

The authors would like to thank Wen Yah Koo, Susan Schelley and Judi Harrington for their excellent technical assistance and the U.S. National Cancer Institute for supplying MISO and SR-2508.

This investigation was funded by Research Grant No. CA-25990 from the National Cancer Institute.

### REFERENCES

Adams, G. E. (1977) Hypoxic cell radiosensitizers for radiotherapy. In *Cancer: A Comprehensive Treatise*, Vol. 6 (Ed. Becker). New York: Plenum. p. 181.

ADAMS, G. E. (1979) Hypoxic cell radiosensitizers in the future development of radiotherapy. In *Radiosensitizers of Hypoxic Cells* (Ed. Breccia *et al.*). Amsterdam: Elsevier/North Holland. p. 245.

ALLALUNIS, M. J., TURNER, A. R., PARTINGTON, J. P. & URTASUN, R. C. (1979) Effect of misonidazole on human and murine hematopoiesis. *Proc. Am. Assoc. Cancer Res.*, **20**, 82A.

BEDFORD, J. S. & MITCHELL, J. B. (1974) The effect of hypoxia on the growth and radiation response of mammalian cells in culture. *Br. J. Radiol.*, **47**, 687.

BEGG, A. C., FU, K. K., KANE, L. J. & PHILLIPS, T. L. (1980) Single-agent chemotherapy of a solid murine tumor assayed by growth delay and cell survival. *Cancer Res.*, **40**, 145.

BROWN, J. M. (1977) Cytotoxic effects of the hypoxic cell radiosensitizer Ro-07-0582 to tumor cells *in vivo*. *Radiat. Res.*, **72**, 469.

BROWN, J. M. & WORKMAN, P. (1980) Partition coefficient as a guide to the development of radiosensitizers which are less toxic than misonidazole. *Radiat. Res.*, **82**, 171.

CLEMENT, J. J., GORMAN, M. S., WODINSKY, I., CATANE, R. & JOHNSON, R. K. (1980) Enhancement of antitumor activity of alkylating agents by the radiation sensitizer misonidazole. *Cancer Res.*, **40**, 4165.

CONROY, P. J., VON BURG, R., PASSALACQUA, W. & SUTHERLAND, R. M. (1980) The effect of misonidazole on some physiologic parameters in mice. *J. Pharmacol. Exp. Ther.*, **212**, 1.

DISCHE, S., SAUNDERS, M. I., LEE, M. E., ADAMS, G. E. & FLOCKHART, I. R. (1977) Clinical testing of the radiosensitizer Ro-07-0582: Experience with multiple doses. *Br. J. Cancer*, **35**, 567.

GOMER, C. J. & JOHNSON, R. J. (1979) Relationship between misonidazole toxicity and core temperature in C3H mice. *Radiat. Res.*, **78**, 329.

HIRST, D. G. & DENEKAMP, J. (1979) Tumour cell proliferation in relation to the vasculature. *Cell Tissue Kinet.*, **12**, 31.

KOCH, C. J., KRUUV, J., FREY, H. E. & SNYDER, R. A. (1973) Plateau phase in growth induced by hypoxia. *Int. J. Radiat. Biol.*, **23**, 67.

LEFEBVRE, Y. & HESSELTINE, M. C. (1965) The peripheral white blood cells and metronidazole. *J. Am. Med. Ass.*, **194**, 15.

ROIZIN-TOWLE, L. & HALL, E. J. (1978) Studies with bleomycin and misonidazole on aerated and hypoxic cells. *Br. J. Cancer*, **37**, 254.

ROSE, C. M., MILLAR, J. L., PEACOCK, J. H. & STEPHENS, T. C. (1979) The effect of misonidazole on *in vivo* tumor cell kill in Lewis lung carcinoma treated with melphalan or cyclophosphamide. *Conference on Combined Modality Cancer Treatment: Radiation Sensitizers and Protectors.* Key Biscayne, Florida, October 1979.

ROSE, C. M., MILLAR, J. L., PEACOCK, J. H., PHELPS, T. A. & STEPHENS, T. C. (1980) Differential enhancement of melphalan cytotoxicity in tumor and normal tissue by misonidazole. In *Radiation Sensitizers* (Ed. Brady). New York: Masson. p. 250.

TANNOCK, I. F. (1968) The relation between cell proliferation and the vascular system in a transplanted mouse mammary tumour. *Br. J. Cancer*, **22**, 258.

TANNOCK, I. F. (1980) The *in vivo* interaction of anticancer drugs with misonidazole or metronidazole: Cyclophosphamide and BCNU. *Br. J. Cancer*, **42**, 871.

THOMLINSON, R. H., DISCHE, S., GRAY, A. J. & ERRINGTON, L. M. (1976) Clinical testing of the radiosensitizer Ro-07-0582: III. Response of tumours. *Clin. Radiol.*, **27**, 167.

TILL, J. E. & MCCULLOCH, E. A. (1961) A direct measurement of the radiation sensitivity of normal mouse bone marrow cells. *Radiat. Res.*, **14**, 213.

TURNER, A. R., ALLALUNIS, M. J., URTASUN, R. C., PEDERSEN, J. E. & MEEKER, B. E. (1980) Cytotoxic and radiosensitizing effects of misonidazole on hematopoiesis in normal and tumor-bearing mice. *Int. J. Radiat. Oncol. Biol. Phys.*, **6**, 1157.

TWENTYMAN, P. R. (1977) The sensitivity to cytotoxic agents of the EMT6 tumour *in vivo*: Comparison of data obtained using tumour volume measurement and *in vitro* plating. I. Cyclophosphamide. *Br. J. Cancer*, **35**, 208.

TWENTYMAN, P. J. (1979) Timing of assays: An important consideration in the determination of clonogenic cell survival both *in vivo* and *in vitro*. *Int. J. Radiat. Oncol. Biol. Phys.*, **5**, 1213.

TWENTYMAN, P. R. (1981) Modification of tumour and host response to cyclophosphamide by misonidazole and by WR 2721. *Br. J. Cancer*, **43**, 745.

TWENTYMAN, P. R., KALLMAN, R. F. & BROWN, J. M. (1979) The effect of time between X-irradiation and chemotherapy on the growth of three solid mouse tumours. I. Adriamycin. *Int. J. Radiat. Oncol. Biol. Phys.*, **5**, 1255.

TWENTYMAN, P. R., BROWN, J. M., GRAY, J. W., FRANKO, A. J., SCOLES, M. A. & KALLMAN, R. F. (1980) A new mouse tumor model system (RIF-1) for comparison of end-point studies. *J. Natl Cancer Inst.*, **64**, 595.

URTASUN, R. C., BAND, P. R., CHAPMAN, J. D., RABIN, H., WILSON, A. F. & FRYER, C. G. (1977) Clinical phase I study of the hypoxic cell radiosensitizer Ro-07-0582, a 2-nitroimidazole derivative. *Radiology*, **122**, 801.

WHITE, R. A., WORKMAN, P. & BROWN, J. M. (1980) The pharmacokinetics, tumor and neural tissue penetrating properties in the dog of SR-2508 and SR-2555—hydrophilic radiosensitizers potentially less toxic than misonidazole. *Radiat. Res.*, **84**, 542.